# Exogenous corticosteroid-induced modulation of RAAS: potential implications for septic shock biomarker analysis

**DOI:** 10.1186/s13613-025-01567-4

**Published:** 2025-09-25

**Authors:** Thomas Uslar, Benjamin Sanfuentes, Rene Baudrand, Glenn Hernández, Eduardo Kattan

**Affiliations:** 1 Department of Endocrinology, School of Medicine, Pontificia Universidad Católica de Chile, Avenida Diagonal Paraguay 362, Santiago, 6510260 Chile; 2 Centro Traslacional de Endocrinología UC (CETREN-UC), Santiago, Chile; 3 Departamento de Medicina Intensiva, Facultad de Medicina, Pontificia Universidad Católica de Chile, Avenida Diagonal Paraguay 362, Santiago, 6510260 Chile

To the Editor,

We read with great interest the recent publication by Benaroua et al., titled *“Alterations in the renin-angiotensin system during septic shock”*, in *Annals of Intensive Care* [1]. The authors provide valuable insights into the dynamic imbalance between classical and alternative renin-angiotensin-aldosterone (RAAS) pathways during septic shock, notably the elevated Ang I/Ang II and Ang-(1–7)//Ang II ratios, reduced circulating ACE activity, and the concurrent rise in ACE2 activity and DPP3 levels. These findings contribute significantly to our understanding of RAAS dysregulation in the critical care context.

We would like to highlight to an important pre-analytical consideration that may influence the interpretation of the RAAS profile in this cohort. Specifically, the concurrent administration of hydrocortisone and fludrocortisone to 75% of the study patients may have introduced exogenous modulation of the RAAS system that was not fully addressed. These corticosteroids possess distinct, yet overlapping, biological effects on renin-angiotensin homeostasis and blood volume regulation.

At high pharmacological doses, hydrocortisone can exceed the protective enzymatic barrier of 11β-hydroxysteroid dehydrogenase type 2 (11β-HSD2), which normally inactivates cortisol within mineralocorticoid-sensitive tissues, mainly in the kidney. When this enzymatic barrier is overwhelmed, hydrocortisone can modulate renin and aldosterone secretion through mineralocorticoid receptors (MR) [2–4],. This MR activation will enhance sodium reabsorption in the kidney, potentially suppressing renin and aldosterone release through a negative feedback loop. Fludrocortisone, a synthetic corticosteroid with high mineralocorticoid potency, further enhances this MR activation and will have a combined effect in suppression of renin secretion [5]. The observed hypoaldosteronism in the study, therefore, may reflect MR-mediated suppression of the renin-aldosterone axis rather than intrinsic adrenal dysfunction, that will classically have low aldosterone and high renin levels.

Moreover, glucocorticoids, particularly in an acute administration [6], promote hepatic angiotensinogen synthesis, which may elevate Ang I concentrations independent of renin activity [7]. Simultaneously, emerging data suggest that glucocorticoids can upregulate ACE2 expression and activity, promoting the shift toward the “alternative” RAAS pathway and potentially elevating Ang-(1–7) levels even in the absence of increased Ang II degradation [8]. Theoretically, these mechanisms, when combined, could modify the equilibrium of circulating RAAS peptides and enzymes, leading to potential confounding in the interpretation of endogenous RAAS physiology during sepsis. These exogenous corticosteroid-induced modulation of RAAS are summarized in Fig. 1.

**Fig. 1:**
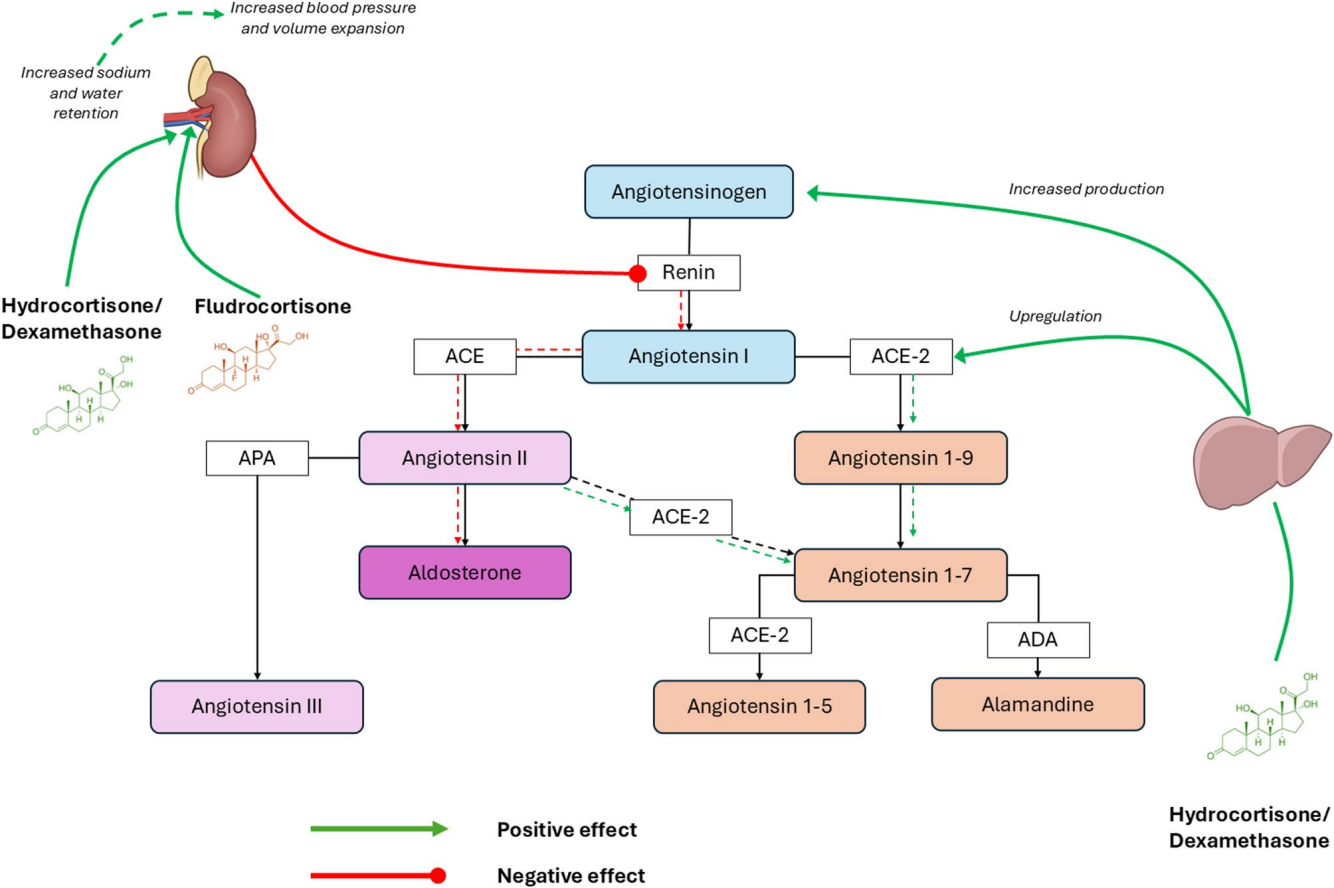
Theoretical effects of exogenous corticosteroid administration on RAAS modulation. ACE: angiotensin converting enzyme; APA: aminopeptidase A; ADA: aspartate decarboxylase

While these aforementioned theoretical interactions have been demonstrated in experimental and other scenarios, there is a significant knowledge gap regarding their relevance in septic shock patients. For instance, in the original cohort, Ang- (1–7) levels decreased over time, suggesting that systemic upregulation did not occur. Whether exogenous corticosteroids significantly alter the RAAS profile in septic shock—or whether this effect is mitigated by factors such as differential steroid potencies or a predominance of tissue-specific rather than systemic effects—remains unclear and warrants further investigation [9, 10]. Ultimately, while the authors correctly excluded prior use of RAAS-modulating medications, the administration of corticosteroid therapy to a large proportion of the cohort limits the interpretation of this study’s results as solely attributable to septic shock, and, thus, we believe it merits further analysis. In future investigations, comparing RAAS profiles in corticosteroid treated versus untreated patients could clarify the extent to which this therapeutic intervention impacts the observed RAAS phenotype. Acknowledging these potential confounders is essential for accurate interpretation of RAAS biomarkers in septic shock and developing treatment strategies accordingly.

## Data Availability

Not applicable.

## Abbreviations

RAAS: Renin-Angiotensin-Aldosterone
11β-HSD2: 1β-Hydroxysteroid Dehydrogenase type 2
MR: Mineralocorticoid Receptors
